# Reply to “Comment on: Optimal Nutritional Status for a Well-Functioning Immune System Is an Important Factor to Protect against Viral Infections. *Nutrients* 2020, *12*, 1181”

**DOI:** 10.3390/nu12082326

**Published:** 2020-08-03

**Authors:** Philip C. Calder, Anitra C. Carr, Adrian F. Gombart, Manfred Eggersdorfer

**Affiliations:** 1 Faculty of Medicine, University of Southampton and NIHR Southampton Biomedical Research Centre, University Hospital Southampton NHS Foundation Trust, Southampton SO16 6YD, UK; 2 Nutrition in Medicine Research Group, Department of Pathology & Biomedical Science, University of Otago, Christchurch 8140, New Zealand; anitra.carr@otago.ac.nz; 3 Linus Pauling Institute, Department of Biochemistry and Biophysics, Oregon State University, Corvallis, OR 97331, USA; adrian.gombart@oregonstate.edu; 4 Department Internal Medicine, University Medical Center Groningen, 9713 GZ Groningen, The Netherlands; m.eggersdorfer@bluewin.ch

We thank Tsoupras and Zabetakis for their interest in our recent publication [1,2]. They provide comments in relation to the omega-3 (*n*-3) fatty acids eicosapentaenoic acid (EPA) and docosahexaenouc acid (DHA). Specifically, they raise points about the role of *n*-3 fatty acids in cardiovascular disease, about effective doses of EPA+DHA in different conditions, and about the chemical form of *n*-3 fatty acid supplements. We agree that these are all important points of on-going discussion, although some are beyond the subject matter of our publication that was specifically about supporting immune function in the context of anti-viral defence. 

As discussed elsewhere [3], the evidence for *n*-3 fatty acids in reducing risk of developing coronary heart disease (CHD) is irrefutable and this is the basis of most current recommendations for intake of these fatty acids by the general population. For example, aggregating data from 16 studies involving over 422,000 individuals showed a 13% risk reduction for combined coronary outcomes for those in the upper tertile of dietary EPA+DHA intake compared with those in the lower tertile of intake [4]. Aggregation of data from 13 studies involving over 20,000 individuals showed risk reductions for combined coronary outcomes of 22%, 21%, and 25% for those in the upper tertile of circulating EPA, DHA, and EPA+DHA, respectively, compared with those in the lower tertile [4]. Combining data from 17 prospective studies show an 18% risk reduction for any CHD event for those with higher dietary intake of EPA+DHA compared to those with lower intake [5]. There were also significant reductions of 23%, 19%, and 47% in the risk for fatal coronary events, coronary death, and sudden cardiac death, respectively [5]. We do agree that findings from randomised controlled trials with EPA and DHA reporting on mortality have been inconsistent [3], but even here, recent studies show benefits. For example, ASCEND reported significantly fewer deaths from vascular events in diabetics receiving EPA+DHA (840 mg/day) than in those receiving placebo, as well as a trend towards reduced risk of death from CHD [6]. VITAL reported a significant reduction in risk of, and death from, either myocardial infarction or CHD in patients receiving EPA+DHA (840 mg/day) [7]. In REDUCE-IT, patients who received 4 g/day EPA-ethyl ester had a significant reduction in the primary outcome (a composite of cardiovascular death, non-fatal myocardial infarction, non-fatal stroke, coronary revascularisation, or unstable angina), the pre-specified secondary outcome, and a whole range of other pre-specified outcomes [8]. A recent meta-analysis included data from 13 randomised controlled trials (127,477 patients; mean follow-up 5 years) [9]: In an analysis excluding REDUCE-IT, *n*-3 fatty acid supplementation was associated with a significantly lower risk of myocardial infarction, CHD death, cardiovascular death, and total cardiovascular disease. These inverse associations for all outcomes were strengthened after including REDUCE-IT [9]. Finally, on the topic of CHD, it is worth noting that in 2017 the American Heart Association reinforced its earlier advice on the role of *n*-3 fatty acids in reducing mortality in patients with prevalent CHD [10].

We fully agree with Tsoupras and Zabetakis that dose of *n*-3 fatty acids is important, and this is evident from the recent meta-analysis of *n*-3 fatty acids and cardiovascular outcomes [9]. Where such studies have been performed in humans, effects of *n*-3 fatty acids on cardiovascular risk factors such as blood triglycerides, blood pressure, platelet reactivity, and inflammation have all been shown to be dose-dependent. There has been some discussion of the dose of EPA and DHA required to induce an anti-inflammatory effect. Certainly, this dose is higher than 250 mg/day and may in fact be more than 2 g/day, as discussed elsewhere [11]. However, this dose is most relevant to a therapeutic effect in a frank inflammatory condition like rheumatoid arthritis. Inflammation is part of host defence, being the initial response of innate immunity to infection. In the context of bacterial or viral infection, dampening inflammation would not make sense and could even be detrimental. Hence, a more modest intake of EPA and DHA is appropriate to supporting host defence, enabling innate immunity, including the inflammatory component, and allowing T and B cells to function well. The dose response relationship between *n*-3 fatty acids and infection has not been studied in humans, but a comprehensive review of animal studies concluded that intakes of EPA and DHA that are in accordance with current recommendations are beneficial against a range of infections and that higher intakes can be detrimental to host defence against some pathogens because of the strong anti-inflammatory effects seen [12].

Tsoupras and Zabetakis mention in passing that different chemical forms of *n*-3 fatty acids (free fatty acids, ethyl esters, triglycerides, phospholipids) might have different biological effects or potencies. Most studies have been performed with triglycerides and ethyl esters; whether they have different effects, perhaps related to bioavailability, is uncertain. Less studied forms of *n*-3 fatty acids such as phospholipids certainly merit more extensive evaluation in human trials. 

Finally, Tsoupras and Zabetakis make a plea for re-evaluating recommendations for *n*-3 fatty acids, a topic we consider beyond our remit. However, it is worth mentioning that setting dietary or nutrient intake recommendations or guidelines for the general population involves many considerations beyond simply the intake(s) required for specific physiological or clinical effects. The situation in “medical nutrition” is different, and it is noteworthy that the recommendations for *n*-3 fatty acids made by the American Heart Association, the European Society of Cardiology, the European Atherosclerosis Society, the American Diabetes Association, and the National Lipid Association are all much greater than those made by national or international agencies (see [3]).
